# First record of the genus *Dialarnaca* Gorochov, 2005 from China, with description of two new species (Orthoptera, Gryllacrididae, Gryllacridinae)

**DOI:** 10.3897/zookeys.558.6165

**Published:** 2016-02-01

**Authors:** Fu-Ming Shi, Xun Bian, Li-Ying Guo

**Affiliations:** 1 The Key Laboratory of Zoological Systematics and Application, College of Life Sciences, Hebei University, Baoding, 071002, China

**Keywords:** Gryllacrididae, Gryllacridinae, new record genus, Dialarnaca, new species, China

## Abstract

In the present paper, *Dialarnaca* Gorochov, 2005 is recorded from China for the first time, with two new species of the genus described, *Dialarnaca longicerca* Shi & Bian, **sp. n.** and *Dialarnaca zhoui* Shi & Bian, **sp. n.** A key and a distribution map of the genus *Dialarnaca*, are provided.

## Introduction

*Dialarnaca* was first proposed by Gorochov (2005) with *Dialarnaca roseola* (type locality: Philippines, Mindoro Island, near Puerto Galera). Currently, except for the type species, no additional species was described (Eades et al. 2015). In this paper, the genus *Dialarnaca* is first recorded from China and two new species, *Dialarnaca longicerca* sp. n. (type locality: Hainan, Changjiang, Bawangling) and *Dialarnaca zhoui* sp. n. (type locality: Yunnan, Jinping, Pinghe), are described. A key to species and a distribution map are provided.

## Material and methods

Specimens in this study were collected from tropical area of southern China. Morphological structures were examined and measured using Leica M205A stereomicroscope. Leica DFC 450 digital imaging system was used to obtain morphological images. The map was drawn using ArcGIS 10.2 based on the occurrence points. The specimens were deposited in the Museum of Hebei University, China.

All measurements of length are in millimetres. The following abbreviations were used for the specimen measurements: body w/wings – the distance from the apex of fastigium verticis to the apex of tegmina; body w/o wings – in male, the distance from the apex of fastigium verticis to the posterior margin of tenth abdominal tergite, in female, the distance from the apex of fastigium verticis to the apex of epiproct; pronotum – the straight-line distance from the anterior margin of pronotum to posterior margin; tegmen – the distance from the base of tegmen to apex; hind femur – the distance from the base of hind femur to the apex of genicular lobe; ovipositor – the distance from the base of subgenital plate to the apex of ovipositor.

## Taxonomy

### Genus Dialarnaca

Taxon classificationAnimaliaOrthopteraGryllacrididae

Gorochov, 2005

Dialarnaca : Gorochov 2005: 821 (English translation by Entomological Review: 931).

#### Type species.

*Dialarnaca roseola* Gorochov, 2005, by original designation.

#### Description.

Body medium, form slender. Eyes kidney-shaped, prominent; ocelli small, inconspicuous. Humeral sinus of pronotum distinct. Tegmina and hind wings developed, far surpassing apices of hind femora; M vein of tegmina simple, free, not united with R vein. Fore coxae with one small spine; fore and middle femora unarmed on ventral surface, tibiae with five pairs of movable ventral spines (including a pair of apical spines); middle tibiae with an inner apical spine on dorsal surface. Hind femora with two rows of minute spines on ventral surface; tibiae with two rows of spines on dorsal surface, subapices with 1 pair of spines on ventral surface, apices with one pair of dorsal spines, the inner one obviously longer than outer one, and two pairs of ventral spines. Second and third abdominal tergites with two rows of transverse stridulatory teeth on lateral margins separately. MALE: ninth abdominal tergite without any hook, centre of posterior margin with a large tubercular process, the angular apex of which slightly directing downwards; tenth abdominal tergite in the form of transverse sclerite, posterior area projecting backwards, membranous, the apex with 1 pair of heavily sclerotized hooks, which curved upwards; subgenital plate variable with well-developed styli; genitalia entirely membranous. FEMALE: posterior area of seventh abdominal sternite projecting backwards; basal area of subgenital plate with numerous rugulae.

#### Diagnosis.

The genus can be identified by the following characters: male ninth abdominal tergite without any process, posterior area of tenth abdominal tergite membranous with one pair of sclerotized apical hooks, which curved upwards. Because only one female is known, the diagnosis of the genus is insufficient.

#### Key to the species of *Dialarnaca*

**Table:** 

1	Apical area of male cerci curly, styli shorter (0.23–0.24 mm), about 3.92–4.43 times shorter than the length of male subgenital plate along the midline	***Dialarnaca longicerca* sp. n.**
–	Male cerci straight, styli longer, about 2.33–2.47 times shorter than the length of male subgenital plate along the midline	**2**
2	Posterior area of male subgenital plate slightly projected, posterior margin shallowly concave in the middle	**Dialarnaca roseola**
–	Posterior area of male subgenital plate trapezoidal projected, posterior margin nearly straight	***Dialarnaca zhoui* sp. n.**

### Dialarnaca longicerca

Taxon classificationAnimaliaOrthopteraGryllacrididae

Shi & Bian sp. n.

http://zoobank.org/329CD64A-FCCF-4FDD-AD33-84D37D974C72

Map 1 , Figs 1–11 , 18–19

#### Type material.

Holotype: male, pinned, China, Hainan, Changjiang, Bawangling, 26 May 2014, coll. by Jiao Jiao. Paratypes: 1 male and 1 female, pinned, China, Hainan, Changjiang, Bawangling, 13 July 2010, coll. by Ming Qiu and Rui-Lian Li.

#### Diagnosis.

This species differs from the *Dialarnaca roseola* Gorochov, 2005 in body green, male cerci longer and apical area curled, posterior margin of male subgenital plate slightly concave in the middle, styli shorter.

#### Description.

**Figures 1–11.**
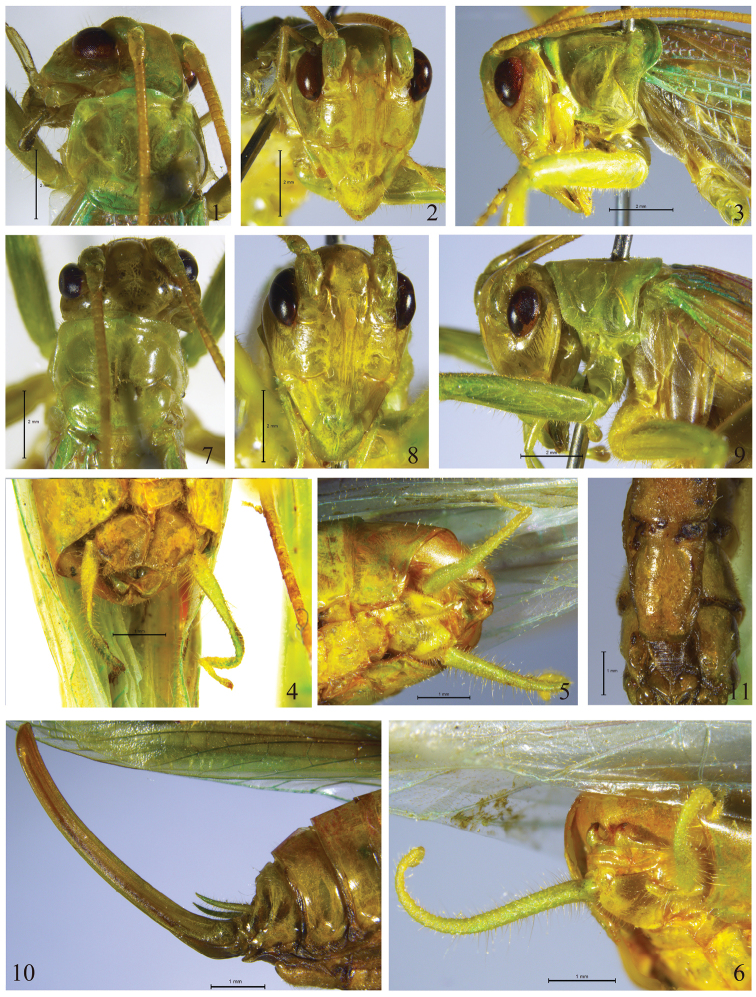
*Dialarnaca longicerca* sp. n.: **1–6** male **7–11** female. **1, 7** head and pronotum in dorsal view **2, 8** head in frontal view **3, 9** head and pronotum in lateral view **4, 11** apex of abdomen in ventral view **5, 6** apex of abdomen in ventro-lateral view **10** apex of abdomen in lateral view.

Male. The following characters are in addition to those given in the generic description. Fastigium verticis broad, about 1.5–1.7 times as wide as scape (Fig. 2). Eyes ovoid; ocelli inconspicuous. Scape about as long as length of eyes, pedicel approximately half as long as scape (Fig. 2). Anterior margin of pronotum projected in the middle, posterior margin almost truncate, lateral lobes longer than high (Figs 1, 3). Hind femora with 5–6 spines on internal margin of ventral surface, external margin with 6–8 spines; tibiae with 5–6 spines on internal margin and seven spines on external margin of dorsal surface. Apical area of ninth abdominal tergite with 1 tubercular process in the middle, which slightly directing downwards; posterior margin of tenth abdominal tergite with one pair of long triangular hooks in the middle, which curved upwards, its apices directing forwards (Figs 4–6, 18–19). Cerci about 4.3–4.4 mm, longer than in other species of the genus, apical area curly, apices obtuse (Fig. 5). Subgenital plate broader than long, anterior margin nearly straight, posterior margin slightly concave in the middle; styli about 0.23–0.24 mm, shorter than in other species of the genus, cylindrical, located on lateral margins of subgenital plate near apex (Fig. 4).

Female. Differs from male in following characters: cerci slender, apices acute. Seventh abdominal sternite long, both sides of four fifths apical area slightly concave, posterior area projecting backwards, centre of posterior margin slightly concave (Figs 10–11). Subgenital plate longer than broad, basal area semimembranous, with abundant fine stripes, posterior margin obtuse-angular. Ovipositor longer than hind femora, distinctly upcurved, apices obliquely roundly cutting (Fig. 10).

Coloration. Body green. Eyes black-brown. Apices of hooks of tenth abdominal tergite blackish.

#### Measurements

**(mm).** Male: body w/wings 33.2–34.0, body w/o wings 18.7–20.5, pronotum 4.5–5.0, tegmen 28.7–29.2, hind femur 11.8–12.4; female: body w/wings 34.0, body w/o wings 21.2, pronotum 5.3, tegmen 28.0, hind femur 11.7, ovipositor 14.0.

#### Distribution.

China (Hainan).

**Map 1.**
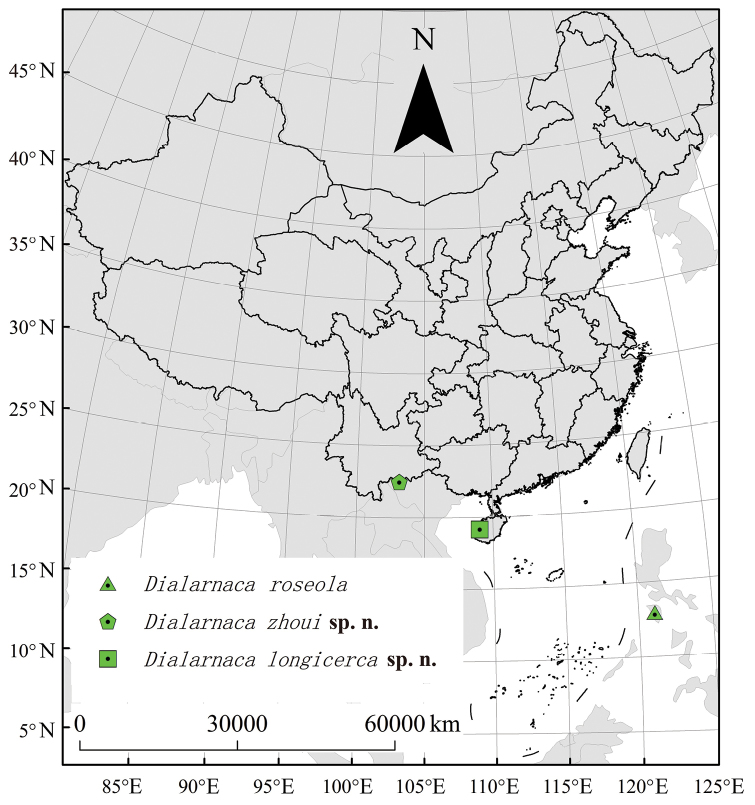
Distribution of *Dialarnaca* species.

#### Etymology.

The name is derived from the longer cerci of male.

### Dialarnaca zhoui

Taxon classificationAnimaliaOrthopteraGryllacrididae

Shi & Bian sp. n.

http://zoobank.org/085D18ED-EC36-490F-8786-BB88548DA126

Map 1 , Figs 12–17 , 20–21

#### Type material.

Holotype: male, pinned, China, Yunnan, Jinping, Pinghe, 12 June 2009, coll. by Fu-Ming Shi.

#### Diagnosis.

This species differs from *Dialarnaca roseola* Gorochov, 2005 and *Dialarnaca longicerca* Shi & Bian, sp. n. by: the posterior area of male subgenital plate trapezoidal projected, styli located on lateral margins near middle area of subgenital plate. In *Dialarnaca roseola*, posterior area of male subgenital plate slightly projected, centre of which with 1 shallow concavity. It can be distinguished from *Dialarnaca longicerca* sp. n. by the shape of male cerci and subgenital plate.

#### Description.

**Figures 12–17.**
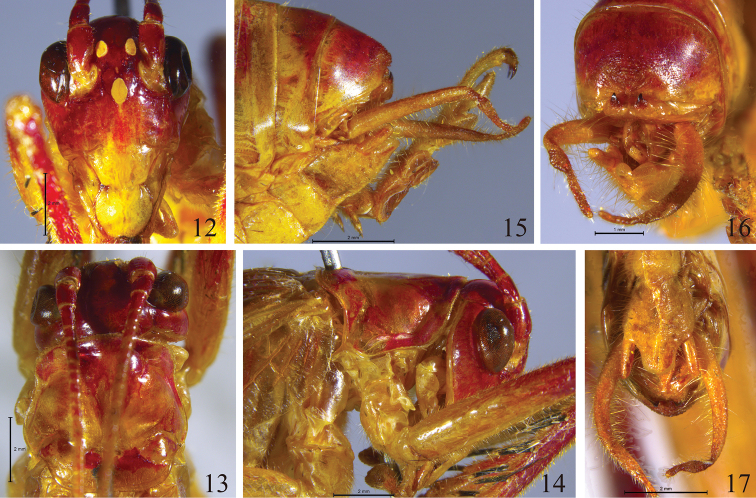
*Dialarnaca zhoui* sp. n., male. **12** head in frontal view **13** head and pronotum in dorsal view **14** head and pronotum in lateral view **15** apex of abdomen in lateral view **16** apex of abdomen in apical view **17** apex of abdomen in ventral view.

Male. Fastigium verticis broad, about 1.5 times as wide as scape. Eyes ovoid; ocelli conspicuous. Scape about three-quarters length of eye, pedicel about half as long as scape (Fig. 12). Anterior margin of pronotum slightly projected, posterior margin almost truncate, ventral margin of lateral lobes undulated (Figs 12–13). Hind femora with 7–9 pairs of spines on ventral surface, tibiae with 4–5 pairs of spines on dorsal surface. Posterior margin of ninth abdominal tergite projecting backwards, centre tubercular; posterior margin of tenth abdominal tergite with 1 pair of short, triangular hooks in the middle, the apices slightly directing forwards in lateral view (Figs 15–16, 20–21). Cerci approximately 3.9 mm, conical, nearly straight, apices obtuse (Figs 16–17). Subgenital plate longer than broad, anterior margin almost straight, posterior area obviously projecting backwards, nearly trapezoid, posterior margin almost straight; styli long (about 0.94 mm), cylindrical, located on lateral margins near middle area of subgenital plate (Fig. 17).

**Figures 18–21.**
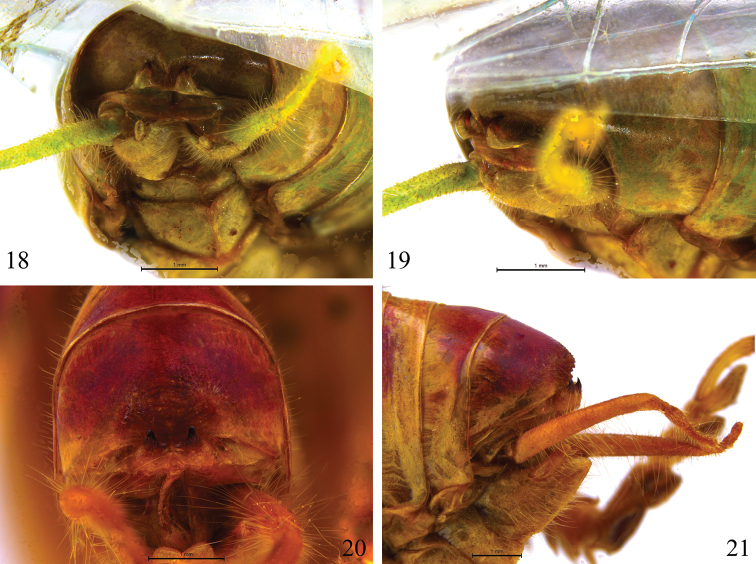
Hooks of male tenth abdominal tergite in *Dialarnaca* spp. **18–19** *Dialarnaca longicerca* sp. n. **20–21** *Dialarnaca zhoui* sp. n. **18, 21** apical view **19, 21** lateral view.

Female. This species is known only from the holotype.

Coloration. Body reddish yellow. Labrum yellow, eyes brown, ocelli yellow. Spines of all legs black.

#### Measurements

**(mm).** Male: body w/wings 42.0, body w/o wings 23.7, pronotum 6.5, tegmen 35.5, hind femur 18.3.

#### Distribution.

China (Yunnan).

#### Etymology.

This species is named in honour of Dr. Shan-Yi Zhou who provided much assistance in collecting specimens.

## Supplementary Material

XML Treatment for Dialarnaca

XML Treatment for Dialarnaca longicerca

XML Treatment for Dialarnaca zhoui
